# Topical Anti-Inflammatory Activity of Oil from* Tropidurus hispidus* (Spix, 1825)

**DOI:** 10.1155/2015/140247

**Published:** 2015-11-18

**Authors:** Israel J. M. Santos, Gerlânia O. Leite, José Galberto M. Costa, Romulo R. N. Alves, Adriana R. Campos, Irwin R. A. Menezes, Francisco Ronaldo V. Freita, Maria Janeth H. Nunes, Waltécio O. Almeida

**Affiliations:** ^1^Laboratory of Zoology, Regional University of Cariri (URCA), Pimenta, 63105-000 Crato, CE, Brazil; ^2^Program of Post-Graduation in Pharmacology, Federal University of Santa Maria, Campus Camobi, 97105-900 Santa Maria, RS, Brazil; ^3^Laboratory of Natural Products Research, Regional University of Cariri (URCA), Pimenta, 63105-000 Crato, CE, Brazil; ^4^Department of Biology, Paraiba State University (UEPB), 58429-500 João Pessoa, PB, Brazil; ^5^Programa de Pós Graduação em Etnobiologia e Conservação da Natureza, Departamento de Ciências Biológicas, Universidade Federal Rural de Pernambuco, Rua Dom Manoel de Medeiros s/n, Dois Irmãos, 52171-900 Recife, PE, Brazil; ^6^Universidade de Fortaleza, Avenida Washington Soares 1321, 60811-905 Fortaleza, CE, Brazil; ^7^Laboratory of Pharmacology and Medicinal Chemistry, Regional University of Cariri (URCA), Pimenta, 63105-000 Crato, CE, Brazil; ^8^Laboratory of Carcinology, Regional University of Cariri (URCA), Pimenta, 63105-000 Crato, CE, Brazil; ^9^Department of Nursing, Regional University of Cariri, 63105-000 Crato, CE, Brazil

## Abstract

*Tropidurus hispidus* has been used in traditional medicine in several regions of Northeastern Region of Brazil. Its medicinal use involves the treatment of diseases such as warts, sore throat, tonsillitis, chicken pox, varicella, measles, asthma, alcoholism, and dermatomycosis. The present study evaluated the topical anti-inflammatory activity of* Tropidurus hispidus* fat in treating ear edema in an animal model. Oil from* T. hispidus* (OTH) was evaluated on its effect against experimental inflammation in mice. OTH was extracted from body fat located in the ventral region of* Tropidurus hispidus* using hexane as a solvent. We used the model of mouse ear edema induced by phlogistic agents, croton oil (single and multiple applications), arachidonic acid, phenol, capsaicin, and histamine, applied into the right ears of animals pretreated with acetone (control), dexamethasone, or OTH. OTH inhibited the dermatitis induced by all noxious agents, except capsaicin. This effect may be related to the fatty acids present in OTH.

## 1. Introduction

The term “Zootherapy” refers to the use of animals and products derived from animals for the treatment of diseases and health conditions [1]. In modern society, zootherapy constitutes an important alternative among many other practiced therapies in the whole world [2, 3]. The high biological and cultural diversity of Brazil translates into a richness of knowledge and traditional practices including the use of animals for medicinal purposes. According to Ferreira et al. [4], a high number of species are used for the treatment of diseases. Alves et al. [5] reported that at least 354 species of medicinal animals are used in Brazil, of which 96% are captured in the field and 21% are mentioned on one or more lists of animals at risk of extinction.

Despite the frequent use of zootherapeutic products in Brazilian traditional medicine, there are still few laboratory studies examining their efficacy [6, 7]. Body fat (lard) and skin are the parts mostly used and they have been traditionally prescribed for the treatment of osteoporosis, rheumatism, arthritis, osteoarthritis, ulcers, gastritis, and inflammatory conditions. These diseases involve an inflammatory process, which suggests a potential use as anti-inflammatory agents, making the evaluation of zootherapeutics important for these purposes [8].

The lizards of the genus* Tropidurus* occur in continental South America, in east and west of the Andes and Galapagos Islands. They are found in savannas, cerrado, caatinga, forests, and plains habitats of South America [9] and inhabit 14 Brazilian states, mainly in the Northeast Region [10, 11].

*Tropidurus hispidus* is one of the species of lizards utilized in Brazilian popular medicine [12] in the treatment of warts [13], sore throat, tonsillitis, and pharyngitis [12]. Santos et al. [14] determined the biological activity of extracts of the skin and decoctions and found that* T. hispidus* showed antibacterial activity when combined with aminoglycosides. Additionally, prospecting studies revealed the presence of alkaloids [14]. On the other hand, besides being used against illnesses of bacterial origin, these lizards are also used in treatment of diseases of inflammatory character [12], although studies analyzing their efficacy have not yet been done. Therefore, the aim of the present work was to evaluate the topical anti-inflammatory activity of the fat of* T. hispidus* in mouse ear edema models.

## 2. Materials and Methods

### 2.1. Zoological Material

A total of 51 lizards of the species* Tropidurus hispidus* were collected (Permission of collection: 154/2007 number 23544-1 process number 17842812) in the municipality of Crato (S 7°14′ and W 39°24′), Ceara, Brazil, in April 2010. They were caught manually with slings and a 4-mm air gun and by active collections combing environments where these animals can occur. Once collected, the lizards were euthanized by freezing and dried at 70°C in drying oven to obtain decoctions and extracts. Voucher specimens were fixed in 70% alcohol and deposited in the collection of Zoology Department of Universidade Regional do Cariri (LZ-URCA-847).

### 2.2. Animals

We used* Swiss* mice (*Mus musculus*), 20–30 g, of both sexes, acclimated at mean temperature of 22°C (±3°C), kept in a 12-hour light/dark cycle, with feed and water* ad libitum*. Animals were donated by the Biotério da Faculdade de Medicina de Juazeiro do Norte (FMJ) (Protocol Number 2009-0319 CEP) and monitored in the Biotério Experimental of Universidade Regional do Cariri (URCA), in accordance with the guidelines and procedures for biosafety of animal houses [15, 16] and bioethics [17].

### 2.3. Chemicals

Arachidonic acid (AA), croton oil, capsaicin, histamine, and indomethacin were obtained from Sigma Chemical Co. (St. Louis, USA). Dexamethasone (Decadron) was acquired from Aché (Brazil). Acetone, ethanol, and hexane of analytical grade were purchased from Dinâmica (Brazil). Ketamine and Xylazine were acquired from Syntec (Brazil).

### 2.4. Obtention of Oil of* Tropidurus hispidus* (OTH)

The oil was extracted from fat concentrated in the ventral region. The extraction was done by a Soxhlet apparatus utilizing hexane as the solvent for a period of 4 hours. The oil was dried in a water bath at 70°C for 2 hours followed by cooling and storing in a freezer for future analyses.

### 2.5. Ear Edema Induced by a Single Application of Croton Oil

Groups of mice (*n* = 7/each) had their right ear treated topically with 20 *μ*L of acetone, 100 mg/mL indomethacin (2 mg/ear), 4 mg/mL dexamethasone (0.08 mg/ear), 100, 200, and 400 mg/mL OTH in acetone (1, 2 and 4 mg/ear, resp.), or crude OTH (20 *μ*L = 13 mg OTH). After 15 minutes, croton oil (5% v/v, 20 *μ*L) in acetone was applied topically on the right ear and 20 *μ*L of acetone (vehicle) on the left ear. After 6 hours, the animals were euthanized and discs with a thickness of 6 mm were obtained for evaluation [18].

### 2.6. Ear Edema Induced by Multiple Applications of Croton Oil

To evaluate the anti-inflammatory effect of OTH in a subacute inflammatory process, already established, we utilized a model with multiple applications of croton oil. An inflammatory process was induced in mice (*n* = 6/group) by the application of croton oil (5% v/v, 20 *μ*L) in acetone on alternate days, for 9 days. OTH (13 mg/ear) and dexamethasone (0.1 mg/ear, positive control) were applied topically for 4 days (twice a day) 96 hours after the first application of croton oil. On the 9th day, animals were euthanized and discs with a thickness of 6 mm were obtained for evaluation [19]. Edema was evaluated daily.

### 2.7. Ear Edema Induced by Application of Phenol, Capsaicin, and Arachidonic Acid

The inflammatory process was induced in mice (*n* = 7/group) by applying on the outer and inner surfaces of the right ear 20 *μ*L of the following phlogistic agents: 10% phenol (v/v); 0.01 mg/*μ*L capsaicin or 0.1 mg/*μ*L arachidonic acid (AA) diluted in acetone. The right ear was treated topically with, 15 minutes before the application of phlogistic agents, OTH (20 *μ*L, pure), acetone (20 *μ*L, negative control), indomethacin (100 mg/mL, positive control for AA), or dexamethasone (4 mg/mL, positive control for phenol and capsaicin). Edema was evaluated 1 h after application of AA and phenol and 30 minutes after application of capsaicin. [20–22].

### 2.8. Ear Edema Induced by Subcutaneous Injection of Histamine

Initially the animals (*n* = 7/group) were anesthetized with 10 mg/kg ketamine hydrochloride and 10 mg/kg xylazine hydrochloride. Next, the animals were topically pretreated with saline (20 *μ*L), dexamethasone (4 mg/mL; 0.08 mg/ear), or pure OTH (13 mg/ear). After 30 minutes, histamine (5 *μ*L, 100 mg/mL in saline) was administered subcutaneously using a 29 G hypodermic needle, in the ventral region of the right ear of the mice. The left ear received the same volume of saline (intradermal). 2 hours later, animals were euthanized and 6-mm discs were obtained from the ears for evaluation [21].

### 2.9. Quantification of Ear Edema

Edema was determined according to the weight obtained due to the inflammatory response. At the end of the period of exposure to each phlogistic agent, mice were euthanized by cervical dislocation and 6-mm discs were obtained from right and left ears with the use of a hole-punch and weighed on a precision balance (Mettler Toledo AB204). Ear edema was determined by the difference in weight (in mg) of the section removed from the right ear (treated with phlogistic agent) and the weight (in mg) of the section removed from the left ear (treated with vehicle used for dilution of irritant).

The mean percentage inhibition of edema (%) was calculated using the following formula: inhibition (%) = [MPE_cont_ − MPE_treat_/MPE_cont_] × 100. MPE_cont_ is the mean percentage of edema of negative control group (treated with saline) and MPE_treat_ is the mean percentage of edema of group subjected to treatment with OTH or standard drug.

### 2.10. Statistical Analysis of Data

The values obtained were expressed as mean ± standard error (SEM). Differences obtained between groups were subjected to one-way analysis of variance (ANOVA) followed by the Student-Newman-Keuls test or two-way ANOVA followed by the Bonferroni test (edema induced by multiple applications of croton oil). The accepted level of significance for the test was *p* < 0.05.

## 3. Results

Croton oil, arachidonic acid, phenol, capsaicin, and histamine elicited a significant inflammatory response, as seen by the increase in weight of the ears treated with the irritants in relation to the ears that received only the corresponding vehicle (acetone).

**Figure 1:**
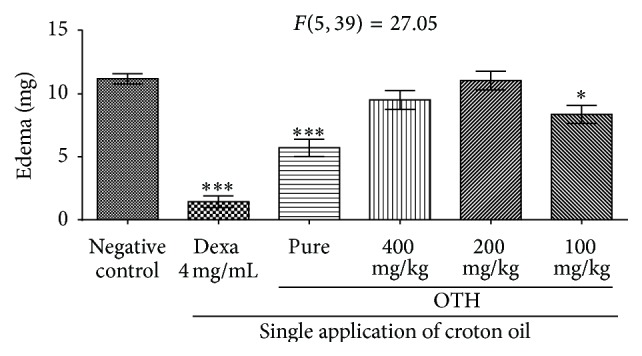
Effect of topical OTH on ear edema induced by single application of croton oil (OC) in Swiss mice. Animals were pretreated with saline in acetone, dexamethasone, OTH (pure, 100, 200, or 400 mg/ear). ANOVA followed by Student-Newman-Keuls test: ^*∗*^ *p* < 0.05 and ^*∗∗∗*^ *p* < 0.001 compared to negative control.

Single application of croton oil induced edema in the right ear of mice (Table 1). The antiinflammatory effect of pure and 100 mg/mL was significant compared to the control group (*p* < 0.001 and *p* < 0.05, resp.) (Figure 1 and Table 1).

**Figure 2:**
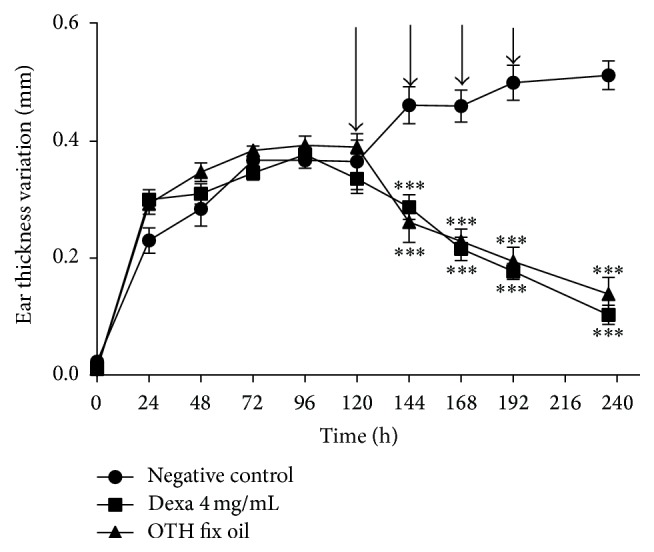
Dose-response curve of effect of OTH on ear edema induced by multiple applications of croton oil. ^*∗∗∗*^ *p* < 0.001 compared to negative control, two-way ANOVA followed by the Bonferroni test.

**Figure 3:**
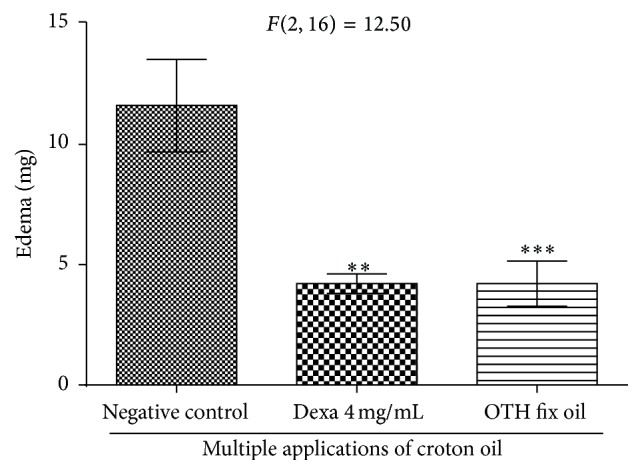
Effect of topical OTH on ear edema induced by multiple applications of croton oil. One-way ANOVA followed by Student-Newman-Keuls test. ^*∗∗*^ *p* < 0.01; ^*∗∗∗*^ *p* < 0.001 compared to negative control.

In the subacute inflammatory model with multiple applications of croton oil, OTH and dexamethasone were applied twice a day for four days (Figure 2, arrows). Negative control showed edema of 11.6 ± 1.9 mg. Dexamethasone and OTH induced a significant decrease to 4.21 ± 0.40 mg (^*∗∗*^ *p* < 0.001, 63.7% inhibition) and 4.21 ± 0.94 mg (^*∗∗∗*^ *p* < 0.001, 63.7% inhibition), respectively (Figures 2 and 3).

**Figure 4:**
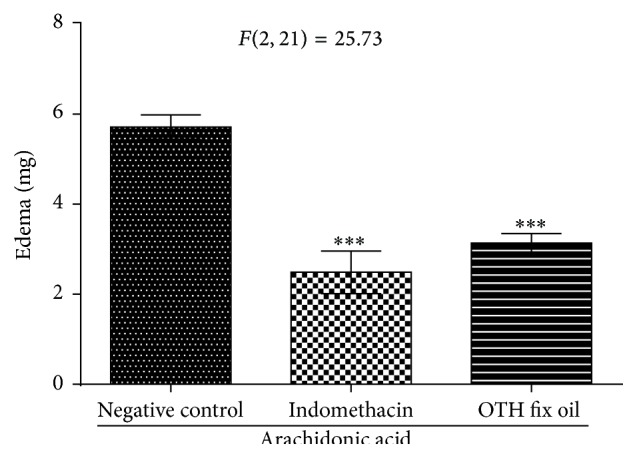
Effect of topical OTH on ear edema induced by arachidonic acid (AA). One-way ANOVA followed by Student-Newman-Keuls test. ^*∗∗∗*^ *p* < 0.001 compared to negative control.

Ear edema induced by AA (5.71 ± 0.26 mg) was also substantially reduced (^*∗∗∗*^ *p* < 0.001) by pure OTH (3.13 ± 0.20 mg, 45.6% inhibition) and indomethacin (2.48 ± 0.47 mg, 57% inhibition) (Figure 4).

**Figure 5:**
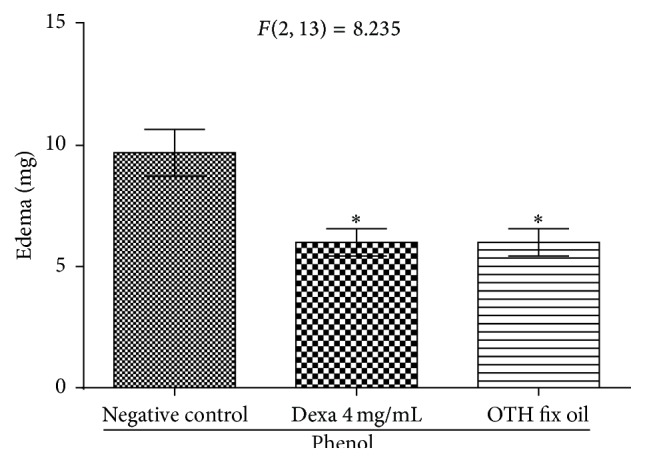
Effect of topical OTH on ear edema induced by phenol. One-way ANOVA followed by Student-Newman-Keuls test. ^*∗*^ *p* < 0.05 and ^*∗∗*^ *p* < 0.01 compared to negative control.

Dexamethasone and OTH inhibited (^*∗*^ *p* < 0.01) the ear edema induced by phenol. The degree of edema of the negative control was of 9.70 ± 0.96 mg, while the groups treated with dexamethasone and OTH were both 6.0 ± 0.56 mg (38.1% inhibition), with the level of significance being *p* < 0.05 for dexamethasone and *p* < 0.01 for OTH (Figure 5).

**Figure 6:**
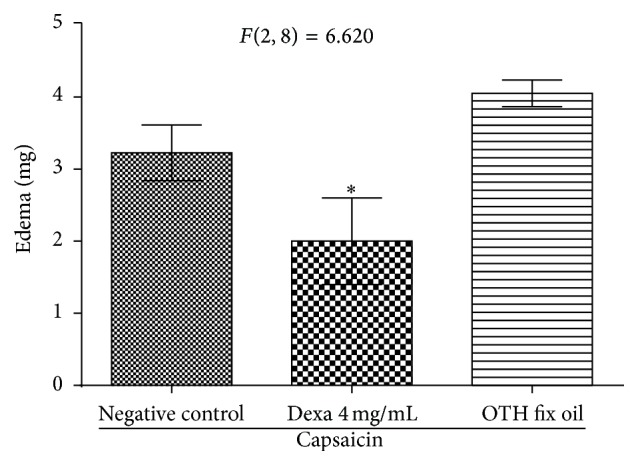
Effect of topical OTH on ear edema induced by capsaicin. One-way ANOVA followed by Student-Newman-Keuls test. ^*∗∗*^ *p* < 0.01 compared to negative control.

Dexamethasone (2.00 ± 0.60 mg; 37.5% inhibition; ^*∗*^ *p* < 0.05), but not OTH, demonstrated an anti-inflammatory effect by reducing the capsaicin-induced ear edema in relation to the negative control (3.22 ± 0.38 mg) (Figure 6).

**Figure 7:**
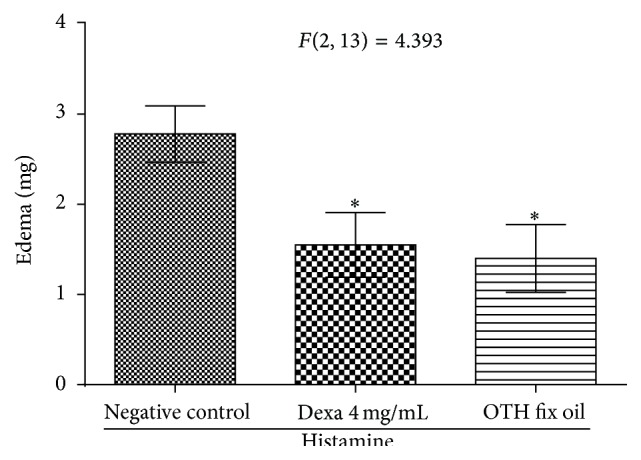
Effect topical of OTH on ear edema induced by the subcutaneous application of histamine. One-way ANOVA followed by the Student-Newman-Keuls test. ^*∗*^ *p* < 0.05 compared to negative control.

OTH (1.40 ± 0.37; 49.6% inhibition) and dexamethasone 1.55 ± 0.35 mg (44.2% inhibition) inhibited (^*∗*^ *p* < 0.05) the ear edema induced by histamine when compared to the control group (2.78 ± 0.31 mg) (Figure 7).

## 4. Discussion

Natural products of animal origin represent an important alternative for the treatment of diseases in people in various parts of the world [23]. According to Coutinho et al. [24], products of animal origin have been methodically tested by pharmaceutical companies as sources of new drugs. Ethnopharmacological studies focusing on remedies of animal origin are very important to indicate the possible therapeutic use of these natural products, as well as possibilities of their pharmaceutical development [25, 26].

Santos et al. [14] performed tests to evaluate microbiological activity of extracts from* T. hispidus* and found that, in combination with aminoglycosides, these extracts produced a significant reduction in the minimum inhibitory concentration (MIC) of the antibiotics, demonstrating the presence of bioactive substances in this species of lizard. In the present work, we showed pharmacological activity of oil from* T. hispidus* (OTH) against experimental topical inflammation. Ferreira et al. [27], studying the medicinal effect of oil from the fat of the lizard* Tupinambis merianae*, showed no antibacterial effect but an effective reduction in topical inflammation induced by the same phlogistic agents used here.

Previous reports related that fatty acids have anti-inflammatory activity, supporting their utilization in the treatment of skin inflammation [28–30]. According to Das [30], fatty acids act directly in inflammatory processes.

We evaluated OTH at concentrations of 100, 200, and 400 mg/mL and the pure oil. Only the pure oil showed anti-inflammatory effect and it was used in the next tests. Subacute inflammation was induced by multiple applications of croton oil. This test simulates the chronic condition in humans and the drug test is administered after the establishment of the inflammatory process, which, according to Alford et al. [31], permits the evaluation of compounds capable of alleviating the subacute inflammation.

According to Green and Shuster [32], anti-inflammatory steroids (corticoids) and lipoxygenase (LOX) inhibitors are active in this model, while cyclooxygenase (COX) inhibitors appear to be inactive. OTH and dexamethasone inhibited the development of edema. When we compare the effect of pure OTH in the models of single and multiple applications of croton oil, we observe that the mean inhibition of edema was greater in multiple applications (63.7%) with a 4-day treatment. This can be explained by the bioavailability of substances that provide local anti-inflammatory action.

Arachidonic acid (AA) is metabolized into various mediators that induce the formation of edema, such as PGE_2_, LTC_4_, and LTD_4_ [33]. According to Griswold et al. [34], the plasma membrane epidermal cells produce AA, which is oxidized to form prostaglandins, leukotrienes, and thromboxanes, responsible for inflammation, as part of the immune response elicited by antigens such as phospholipase A_2_ (PLA_2_). Thus, it is possible to identify, in this model, compounds that inhibit AA metabolism into prostaglandins (PG) and leukotrienes. However, Del Carmen Recio et al. [35] suggested that the edema induced by AA is preferentially a triage model for identifying LOX inhibitors. The nonsteroidal anti-inflammatory drugs (NSAID) inhibit the COX pathway, thereby impeding the synthesis of PG [36]. Thus, the reference drug utilized in this experimental model was indomethacin, an NSAID with anti-inflammatory action related to the nonselective inhibition of the COX isoforms (COX-1 and COX-2) and that effectively reverses edema induced by the topical application of AA, as described by Gabor [22]. Similarly, to indomethacin, OTH also appeared to be effective in inhibiting the formation of edema in this model, significantly decreasing the edema in ears sensitized by AA. According to Carlson et al. [37], models of edema induced by croton oil and AA are extremely useful in the detection of possible COX/LOX inhibitors in vivo. Thus, we believe that OTH is efficient in skin inflammatory disorders, and the use of* T. hispidus* in popular medicine for treating inflammatory diseases could be related to the reduction of the levels of AA metabolites in cutaneous tissue.

OTH did not inhibit the formation of edema induced by the topical application of capsaicin, suggesting that the compounds present in OTH do not appear to act directly on TRPV1 receptors. Studies have demonstrated that the edema induced by the topical application of capsaicin is inhibited by histamine and serotonin antagonists but not by COX inhibitors, such as indomethacin [38].

In the model of edema induced by histamine applied intradermally, the phlogistic agent causes vasodilation and an increase in vascular permeability, causing an edematogenic effect in a few minutes [39]. Prior topical application of OTH significantly inhibited edema induced by histamine.

Pure OTH also significantly reduced edema induced by phenol demonstrating the efficacy of this extract in the treatment of contact dermatitis caused by irritants. In response to exogenous stimuli, such as phenol, keratinocytes produce chemical mediators important in primary contact irritation, including cytokines associated with proinflammatory properties, as IL-1*α*, TNF-*α*, and IL-8 [40, 41]. One of the mechanisms by which phenol triggers cutaneous irritation is the rupture of the plasma membrane of keratinocytes through a direct effect, resulting in the release of preformed IL-1*α*, besides other inflammatory mediators such as the AA metabolites and reactive oxygen species (ROS).

However, despite the fact that the inflammatory response can be triggered by different routes, both models (histamine and phenol) share the involvement of AA metabolites and ROS. Therefore, we found that OTH could exert its anti-inflammatory effect by acting on these mediators, as demonstrated in the AA model. In view of the models of ear edema induced by croton oil, AA, capsaicin, histamine, and phenol, we found that OTH possibly has similar mechanism as drugs that reduce the production of AA metabolites. It is therefore suggested that the antiedematogenic action of OTH is linked to the factors that alter the production of inflammatory eicosanoids, where its action can be through the inhibition of the enzymes COX and LOX or the production of anti-inflammatory eicosanoids (PGE1, lipoxins, or others).

The anti-inflammatory activity of natural products obtained from animals has been proven in other studies. Yoganathan et al. [42] found that the oil from the bird* Dromaius novaehollandiae* promoted a 70% reduction of the ear inflammation in rats. Falodun et al. [43] observed the efficacy of fat from* Boa constrictor* against skin inflammation. These results showed the importance of evaluating more natural products obtained from animals in the treatment of inflammatory diseases.

Considering that animals represent an important source of medications used in traditional medicine, zootherapy has become extremely relevant within a conservationist perspective [44–46]. Alves and Rosa [47] emphasized that the great majority of animals traded for medicinal purposes are wild, making it necessary, most of the time, to kill them to obtain the zootherapeutic products, demonstrating that conservationist measures are needed regarding medicinal species threatened with extinction or that are widely used.

The substitution of threatened medicinal species by other animals with the same therapeutic indications, and not in extinction process, has become a strategy of sustainability and conservation. Sodeinde and Soewu [48] commented that the pressure on threatened species utilized in traditional medicinal formulations could be reduced by their substitution by common species, when suitable, calling attention to sustainability of the substitute species aimed at assuring the viability of its exploitation.* Tropidurus hispidus* can be, in a certain context, considered a substitute species in relation to those threatened, since their existence does not run the risk of extinction and its use in traditional medicine is relatively low. As observed in this study, such species shows significant topical anti-inflammatory activity, and Santos et al. [14] have described its biological activity in combination with aminoglycosides against bacterial strains, thereby making it an alternative in management as a substitute species for those that show the same therapeutic indications and that are threatened.

## 5. Conclusion

The oil derived from the body fat of* T. hispidus* has anti-inflammatory activity against ear edema induced by croton oil, arachidonic acid, histamine, or phenol. The fatty acids present in OTH probably affect arachidonic acid and its metabolites, thereby reducing the production of proinflammatory mediators. In view of the great importance of animals in zootherapy, management and sustainability policies are needed in relation to threatened species.

## Figures and Tables

**Table 1:** Effect of OTH on ear edema induced by single application of croton oil.

Group	Concentration (mg/mL)	Edema (mg)	Inhibition (%)
Negative control	—	11.20 ± 0.39	—
Dexamethasone	4	1.43 ± 0.46^*∗∗∗*^	87.5%
OTH	Pure	5.71 ± 0.68^*∗∗∗*^	49.1%
OTH	100	8.37 ± 0.71^*∗*^	25.0%
OTH	200	11.00 ± 0.73	1.78%
OTH	400	9.51 ± 0.74	15.0%

Values expressed as mean ± SEM (^*∗*^ *p* < 0.05; ^*∗∗∗*^ *p* < 0.001 versus control); (ANOVA and Student-Newman-Keuls test).
